# The costs of repatriating an ill seafarer: a micro-costing approach

**DOI:** 10.1186/s13561-017-0184-0

**Published:** 2017-12-06

**Authors:** Mads D. Faurby, Olaf C. Jensen, Lulu Hjarnoe, Despena Andrioti

**Affiliations:** 1 Centre of Maritime Health and Society, Institute of Public Health, Faculty of Health Sciences, University of Southern Denmark, Niels Bohrs vej 9, 6700 Esbjerg, Denmark; 2 Faurby Consulting, Aebleparken 190, 3rd floor, 5270 Odense N, Denmark

**Keywords:** Local level costing method, Case vignette, Health promotion, Direct cost, Indirect cost, Repatriation

## Abstract

Seafarers sail the high seas around the globe. In case of illness, they are protected by international regulations stating that the employers must pay all expenses in relation to repatriation, but very little is known about the cost of these repatriations. The objective of this study was to estimate the financial burden of repatriations in case of illness. We applied a local approach, a micro-costing method, with an employer perspective using four case vignettes: I) Acute myocardial infarction (AMI), II) Malignant hypertension, III) Appendicitis and IV) Malaria. Direct cost data were derived from the Danish Maritime Authority while for indirect costs estimations were applied using the friction cost approach. The average total costs of repatriation varied for the four case vignettes; AMI (98,823 EUR), Malignant hypertension (47,597 EUR), Appendicitis (58,639 EUR) and Malaria (23,792 EUR) mainly due to large variations in the average direct costs which ranged between 9560 euro in the malaria case and 77,255 in the AMI case. Repatriating an ill seafarer is a costly operation and employers have a financial interest in promoting the health of seafarers by introducing or further strengthen cost-effective prevention programs and hereby reducing the number of repatriations.

## Background

Seafarers are an essential workforce to the global economy with around 1.5 million people working day and night [1], securing transportation of more than 90% of the goods across the globe [2, 3]. The remote character of their working environment defines them as a hard to reach population group [4–6]. This vulnerability of seafarers makes their health and wellbeing a concern and priority in a public health point of view. The current international regulation (Maritime Labour Convention (MLC 2006), states that seafarers must receive equal quality of care as the population on shore [7, 8]. In case of sickness on board, seafarers might find themselves in need of medical evacuation and/or repatriation^1^ [9].

Limited epidemiological research on repatriations is available [3, 10, 11], but suggests that around 1.7% of all deployments ends with repatriation [10], and it is the employer who must pay the costs of repatriation [9], which is likely to be highly expensive. Direct and indirect costs should be taken into consideration. The direct costs includes paying for transportation from the ship to the hospital, hospital admission, medicines, plus transportation to the home country, accommodation salary and sickness benefits during repatriation and illness [9]. The direct costs are often reimbursed by a third party payer to which ship owners pay annual fees in order for their coverage [12]. Indirect costs, such as production loss and the cost of time spent managing the repatriation case, are not reimbursed or insured against. These indirect costs are all held entirely by the employer, which could be as much as ten times the amount of direct costs [13]. Henny et al. in 2013 estimated that the annual costs of evacuation and medical treatment for the shipping industry amount to a total of 760 million euro. Knowing the costs of repatriation for seafarers can provide valuable insights for the employers, who strive to cost containment at all times and maximizing revenues [14]. A cost-of-illness analysis may thus be the first step in an economic evaluation of repatriations to establish strong arguments to promote the health and welfare of seafarers. A micro-level cost analysis of repatriation establishes information regarding the costs of different illnesses and what the major cost drivers are, when seafarers are repatriated [15]. This type of study will provide value to maritime stakeholders and decision makers [16] and eventually enable employers to implement cost effective preventive measures and better integrated care and thereby save money.

The cost of repatriation is an unexplored field and careful attention should be paid to the costing methodology as at the moment there is no golden standard. It is crucial that the cost formula represents the relevant costing categories, is reliable, valid, and user friendly in order to provide useful data and be implemented in the shipping industry and at the ship level [17].

The European Union HealthBASKET project proposed a case vignette method to estimate the costs of treatment. The case vignette is an innovative and novel approach developed to explore resource use and costs.

A case vignette describes a typical patient in regards to diagnosis, age, gender and possible comorbidity and is a retrospective episode-specific costing approach. The approach was applied to the vignette cases – diagnoses – in order to compare the costs of ten different treatments under DRG tariffs across nine European Union countries [15, 18].

Even though research on the evaluation of the financial burden of a range of diseases in different countries and public administration levels is widely available, very limited research concerns the shipping sector.

The objective of this study was to estimate the average total costs of repatriating seafarers based on four case vignettes.

## Methods

Four case vignettes were used (Table 1) based on published epidemiological research, which represent a major disease burden for the seafarers [3, 10].

**Table 1:** Overview of case vignettes

WHO ICD-10 code	Description
IX Diseases of the circulatory system: I21	Acute myocardial infarction – A male aged 45–55
IX Diseases of the circulatory system: I10	Malignant Hypertension – A male aged 45–55
XI Diseases of the digestive system: K35	Acute appendicitis – Male/Female aged 20–30
I Certain infectious and parasitic diseases: B54	B54 Malaria – Male/Female, all age-groups

### Identifying the cases

The primary data source for cost information was the Danish Maritime Authority (DMA). DMA reimburses the ship-owners costs of repatriation and keeps records of the reimbursements made. However, DMA keeps paper archives of all the reimbursement cases, making it highly difficult and time consuming to identify the relevant cases to fit the case vignettes. The identification of the cases to fit the case vignettes were instead derived from Radio Medical Denmark records, which contains information on the date of the call, the expected diagnosis, personal identification number, gender of the seafarer and whether a helicopter emergency medical service or deviation was used to get the seafarer from the ship to shore. For each case vignette, one anonymized match was derived from the Radio Medical Denmark records in the period 2011 to 2013 The survey was approved by the responsible Authority, the Danish Data Protection Agency.

### Establishing cost categories and measuring costs

This study estimates the average total costs of repatriation with an employer perspective based on the local approach [17] and follows guidelines for a costing study for each of the four case vignettes listed above [19]. In every case the timeframe is no longer than 18 weeks since this is the maximum time that is reimbursed by DMA [20, 21].

### Cost formula

The following cost formula was applied to estimate the average total costs of repatriation capturing both the direct and indirect costs of repatriation: *C*_*Total*_ *= C*_*Direct*_ *+ C*_*Indirect*_ *.*

The direct costs of repatriation are related to evacuation via helicopter or ship deviation, further transportation costs such as ambulance and airplane to repatriation country, hospitalization, medication, rehabilitation and sickness benefits, expressed in the formula [22]:

$$ {C}_{Direct}={C}_{Transport}+{C}_{Treatment}+{C}_{Compensation} $$

The indirect costs are expressed as: *C*_*Indirect*_ *= C*_*production loss*_ *+ C*_*recruit*_ *+ C*_*overtime*_ *+ C*_*insurance premium*_ *+ C*_*manage*_ *.*

*Where:*

*C*_*production loss*_: the costs resulting from a slowdown in production.

*C*_*recruit*_: the costs of hiring an additional worker to replace to repatriated seafarer.

*C*_*overtime*_: the costs related to paying overtime to fellow seafarers in order to avoid a slowdown in production.

*C*_*insurance premium*_: the costs related to an increase in insurance premium, which occurs after having the costs related to repatriation reimbursed.

*C*_*manage*_: the costs related to managing the repatriation case e.g. the captain of the vessel must spend time compiling the receipts and claiming reimbursement [17].

This formula is flexible enough to accommodate the data from DMA and company level.

### Identification and classification of resource items and units of resources utilized

A detailed description of the resource utilization of each of the four case vignettes were created and the resource utilization for each case vignette was measured using the local approach [13, 17]. The local approach is characterized by acknowledging that there can be differences in the costs of a service in different cases. The approach involves an assessment of the costs more directly by the company [13], and it is characterized as a micro-costing approach. This method is the most accurate, but also the most time consuming [14]. Each case was presented with the average indirect, direct and total costs based on the data from DMA. Indirect costs, such as insurance premiums, costs related to managing the repatriation case, and replacement costs are not captured in the data from DMA and assumptions in this regard had to be made. We used adjusted aggregate published data, when data were not available.

### Measuring resource units and placing a monetary value on the resource units

The fourth and final step was to measure resource units and place a monetary value on the consumption, taken into consideration both direct and indirect costs.

### Direct costs

The data for estimating the direct costs were retrieved from DMA based on the claims filed by the shipping companies [17]. These claims include fees and charges from hospitals, transportation services, pharmacies, general practitioners and sickness benefits. The costs were all provided in Danish Kroner (DKK). This was converted to euro (EUR) with current exchange rate from the European Central Bank [23]. As the costs occurred in different time periods 2011 to 2013, the figures were adjusted to 2013 prices using the Danish retail price index from Statistics Denmark [24]. The repatriated seafarer was entitled to sickness benefits, which were included as a part of the direct costs. Information about sickness benefits, duration of sick leave and salary was available from DMA. Information about salary used estimations from Marine Insight [25].

### Indirect costs

Less information was available to estimate the indirect costs including productivity losses, management of the case and recruitment of a new seafarer. Assumptions were based on the available literature (Table 2). The friction method was applied to estimate these costs. It was assumed that the friction period lasted 18 weeks, which was the maximum duration that sickness benefits were covered by the DMA [26].

**Table 2:** Summary of cost estimates and assumptions

Category	Description	Estimates in EUR and source of estimations
Direct costs	Average cost per hour of ship diversion (100 ton/24*525 EUR)	2200 per hour of diversion [2]
Direct costs	Average costs per helicopter mission	25,000 [2]
Direct costs	Seafarer wage in the first month of absence	Depends on rank of the seafarer [25]
Indirect costs	Overtime for fellow seafarers 8 h per day spent working overtime	Based on salary [25]
Indirect costs	Replacement costs (1 flight ticket to the vessel)	1500 [22]
Indirect costs	Replacement costs (up front salary to new seafarer 2 months)	Same rank and monthly salary as the repatriated seafarer [25]
Indirect costs	Insurance premium increase [2]	10% of reimbursed costs [2]
Indirect costs	Captain of the vessel managing the case	100 Euro per contact with DMA and Radio Medical (assumption)

## Results

The average total costs of the four case vignettes varied between 23,792 euro for the least expensive – the malaria case vignette – up to 98,823 for the most expensive – the AMI case vignette. Table 3 illustrates the average direct, indirect and total costs of the four case vignettes.

**Table 3:** Overview of the average direct, indirect and total costs of repatriation in 2013 Euro prices

Cost category	AMI	Malignant hypertension	Appendicitis	Malaria
Direct	77,255 (78%)	25,852 (54%)	43,114 (74%)	9560 (40%)
Indirect	21,567 (22%)	21,745 (46%)	15,523 (26%)	14,232 (60%)
Total	98,823 (100%)	47,597 (100%)	58,639 (100%)	23,792 (100%)

### More specifically:

#### Case vignette no. 1

The AMI case vignette was comprised of a male seafarer who was evacuated by helicopter to the port of Bergen, Norway in 2011. The treatment was a percutaneous coronary intervention (PCI) and he was hospitalized for 12 days before returning to his home country, where he received further treatment and hospitalization. The seafarer received sickness benefits for the full period of 18 weeks. The total average cost amounted to 98,823 Euro. The main cost driver for this repatriation case was the treatment costs, which accounted for 44.7% of total costs equaling to 44,174 euro.

#### Case vignette no. 2

The malignant hypertension case vignette was comprised of a male engineer who was evacuated to the port of Shanghai for medical examination due to high blood pressure, in 2013. The ship had to deviate for 6 h to get to the port of Shanghai. The seafarer was found not-fit-for-duty and transported to his country of origin. The data from DMA provided no information on the sickness duration and it was assumed that the seafarer was ill for the full 18 week period that DMA reimburses. The average total costs were estimated at 47,597 euro. The main cost driver was the deviation cost, which accounted for 13,200 euro (27.7%) [25].

#### Case vignette no. 3

The appendicitis case vignette was comprised of the ships cook turning ill with severe abdominal pain in 2011. She was evacuated to the port of Malaga. The ship had to deviate for 12 h to get to the port of Malaga, where the seafarer was transported to the hospital for an appendectomy. As illustrated in Table 3 the majority of the average total costs were attributed to the direct costs. Of the total amount of 58,639 euro, about half (46.5%) was attributed to the ship deviation, with next higher the hospitalization cost at (17.7%).

#### Case vignette no. 4

The malaria case vignette comprised of a deck officer. The seafarer was evacuated to the port of Norfolk Virginia after a rapid malaria test shown a positive result and from the port to the hospital for further diagnosis and treatment. A boat service was utilized to get the seafarer from the vessel to shore. The patient was initiated on malaria treatment and discharged from the hospital the following day. The seafarer was found not-fit-for-duty and had to be repatriated back to his home country. The total average cost amounted to 23,792 euro, with the main single cost driver the cost of a new recruitment, which accounted for 49.7% of the average total costs of this repatriation case equivalent to 11,825 euro.

#### Sensitivity analysis

A sensitivity analysis was carried out for all the four cases in order to investigate the robustness of the cost categories. The sensitivity analysis addressed the uncertainties regarding the cost estimates and assumptions [14]. The sensitivity analysis was carried out with an optimistic and a pessimistic case scenario. The inputs for the sensitivity analysis are provided in Table 4. The costing categories prone to sensitivity analysis were the cost of transportation, (helicopter or fuel consumption in case of ship deviations) compensation costs to the repatriated seafarer, the cost of managing the repatriation case, insurance premium increase and the cost of recruiting a new seafarer. The reimbursed costs by DMA were not prone to the sensitivity analysis, since no uncertainty surrounded these estimates.

**Table 4:** Summary of cost estimates and assumptions

Category	Description	Optimistic assumption/estimate	Base case assumption/estimate	Pessimistic assumption/estimate
Direct costs	Average cost per ship diversion	1100 per hour of diversion (assumption)	2200 per hour of diversion [2]	3300 per hour of diversion (assumption)
Direct costs	Average costs per helicopter mission	9200 [34, 35]	25,000 [2]	37,500 (assumption)
Direct costs	Seafarer wage in the first month of absence	International Transport Workers Federation (ITF) minimal wage [37]	Marine Insight data [25]	PayScale Inc. data [38]
Indirect costs	Overtime	ITFminimal hourly wage [37]	Marine Insight data [25]	PayScale Inc. data [38]
Indirect costs	Replacement costs - 1 flight ticket (assumption)	500	1500	5000
Indirect costs	Replacement costs - salary	Two month salary up front, similar to repatriated seafarer (assumption)		
Indirect costs	Insurance premium increase (assumption)	None	10% of reimbursed costs	20% of reimbursed costs
Indirect costs	Master of the ship managing the case (assumption)	50 Euro per contact with DMA and Radio Medical	100 Euro per contact with DMA and Radio Medical	200 Euro per contact with DMA and Radio Medical

**Fig. 1:**
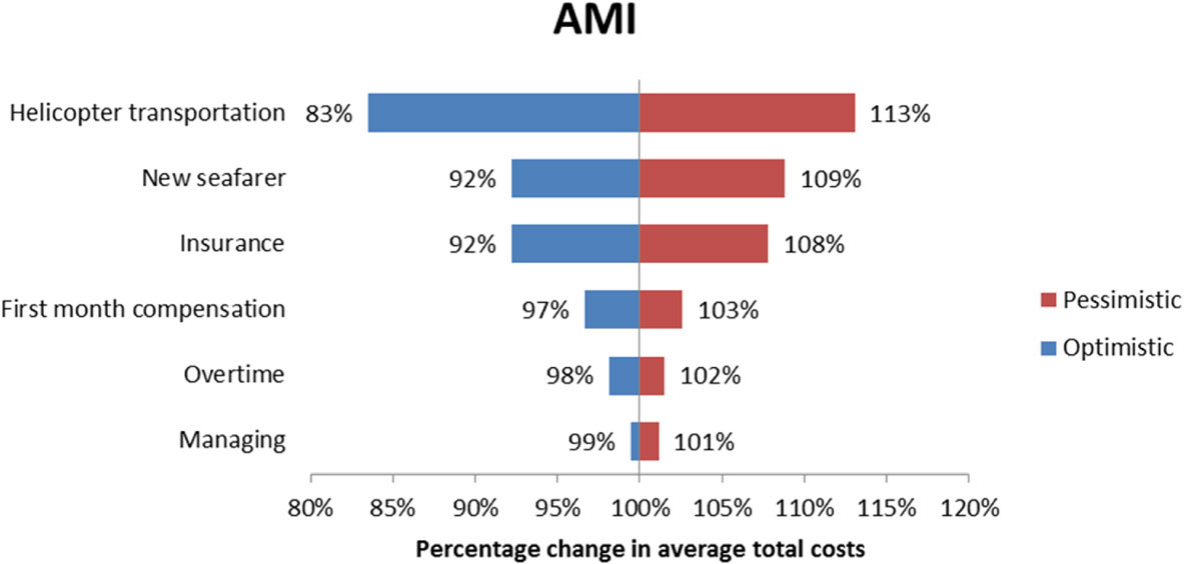
Tornado diagram illustrating the impact of the sensitivity analysis on the average total costs of AMI

**Fig. 2:**
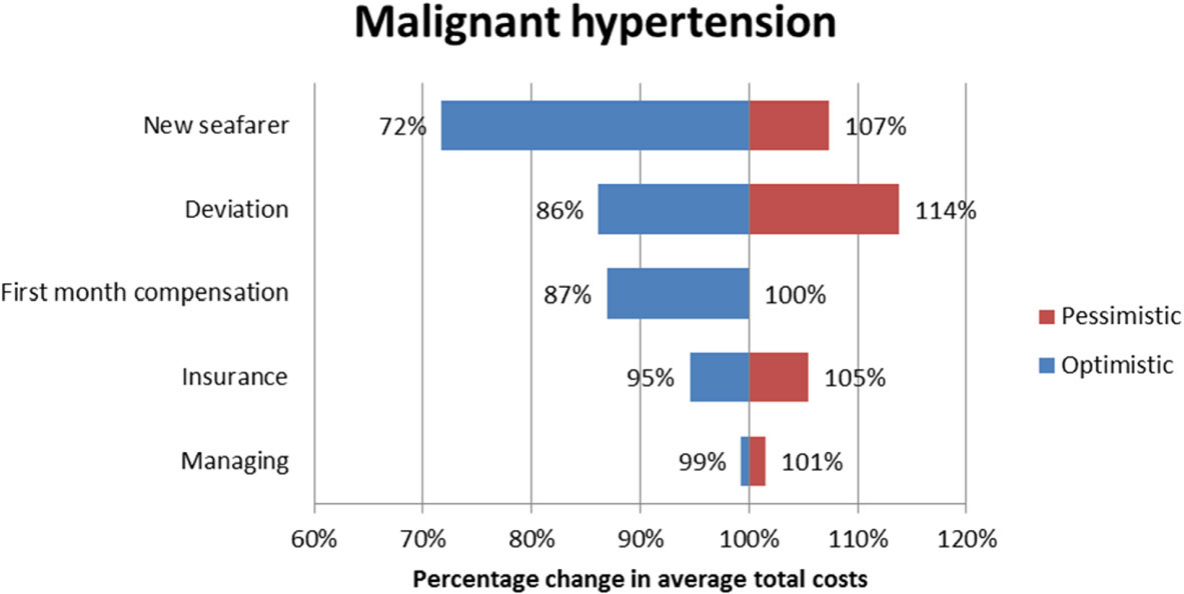
Tornado diagram illustrating the impact of the sensitivity analysis on the average total costs of repatriation due to malignant hypertension

**Fig. 3:**
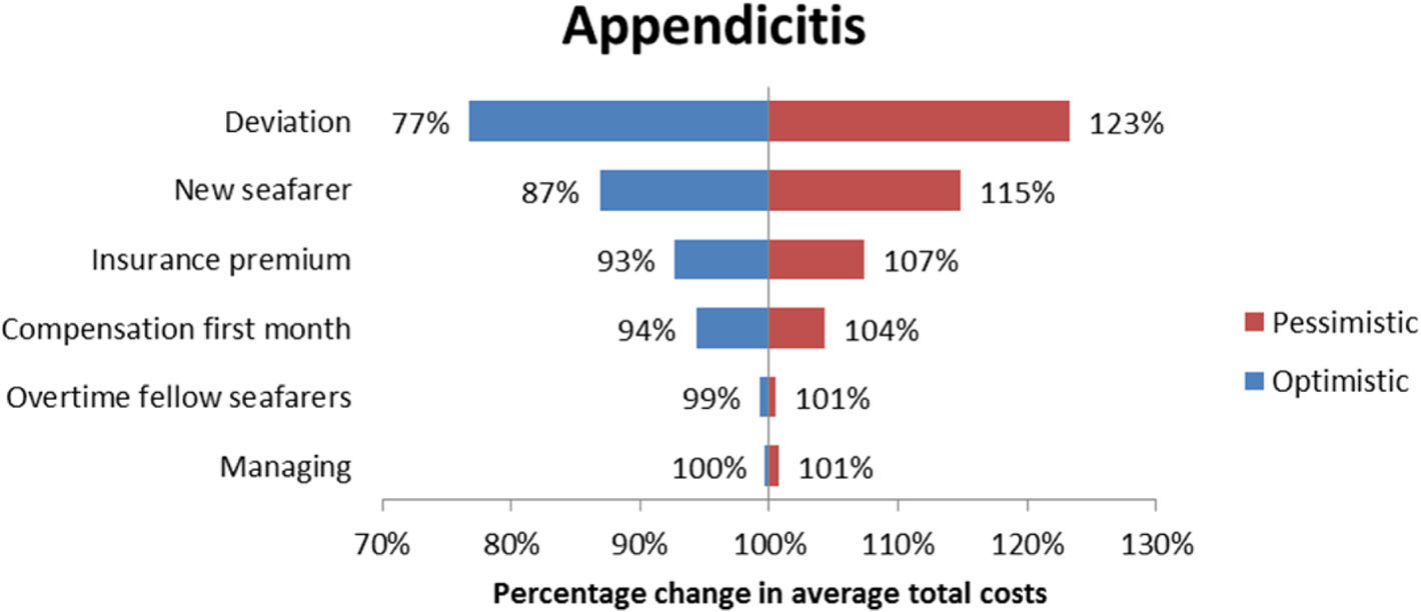
Tornado diagram illustrating the impact of the sensitivity analysis on the average total costs of repatriation due to appendicitis

**Fig. 4:**
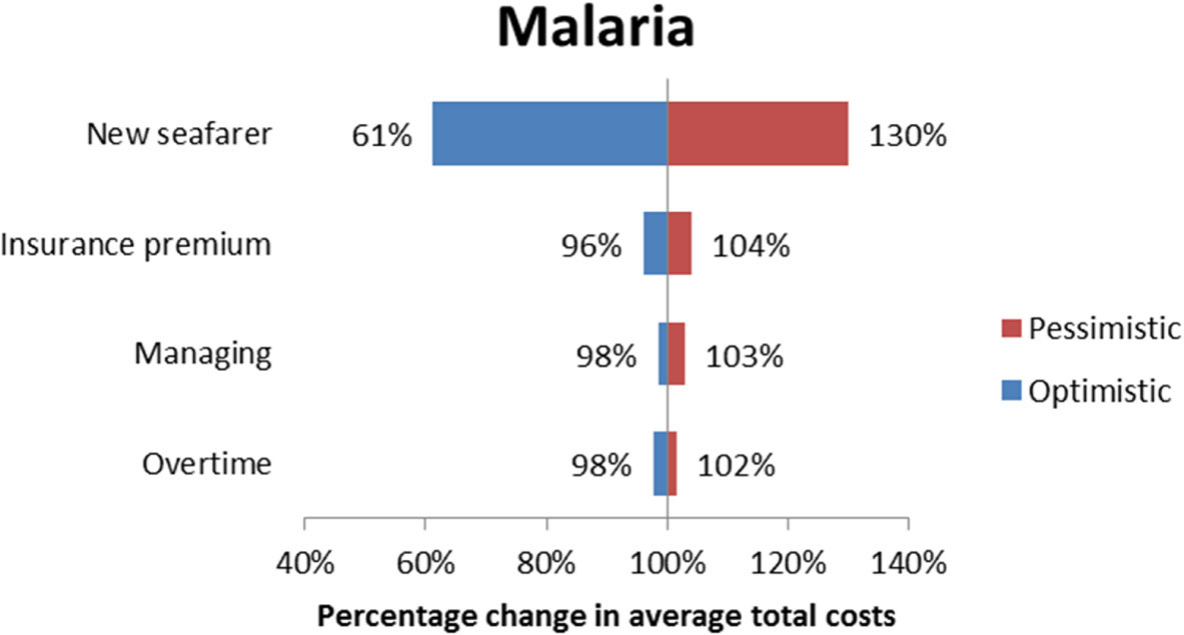
Tornado diagram illustrating the impact of the sensitivity analysis on the average total costs of repatriation for the malaria case vignette

The sensitivity analysis revealed that deviation and evacuation had major impact on the average total costs of repatriation. More specifically, in the appendicitis case the average total costs changed by as much as 23% by applying the pessimistic and optimistic costs for deviation. For the AMI case vignette the tornado diagram (Fig. 1) illustrates the changes in the estimates of the average total costs of repatriation in the optimistic and the pessimistic case scenario. In the optimistic case scenario the reduced costs of helicopter evacuation reduced the estimated average total costs with 17%. The sensitivity analysis for the malignant hypertension case vignette the major cost categories were the costs of recruiting a new seafarer and deviating the vessel. In the optimistic case scenario reductions in the costs of recruiting a new seafarer led to an estimated reduction in the average total costs by 28% and in the pessimistic scenario increases in the costs of deviating the vessel led to an estimated increase in the average total costs by 14% (Fig. 2), similar results were seen in the appendicitis and malaria case vignette (Figs. 3 and 4 respectively). On the contrary, the costing categories compensation and insurance premium increase had an impact on the average total costs of repatriation of less than 10% in both sensitivity analysis scenarios for all the four case vignettes and the same applies to the management of the repatriation case with a no more than 3% impact in any of the scenarios.

## Discussion

Limited published literature is available on the topic of repatriating seafarers [2], showing the relevance and meaningfulness of the estimates provided in this study. This survey applied a retrospectively micro-costing approach to estimate the financial burden of repatriation. Costs were measured by a local approach using company level data [13]. In some instances, such as the helicopter evacuation and deviation costs published data were used [2, 27]. Cost data were attributed to four cases vignettes. The case vignette approach assigns values to resources used in diagnoses – “vignette case” – and in this way comparisons among different countries can be made. In this study, the data yielded large variations in the average total costs of repatriation, where the costs ranged between 23,792 and 98,823 euros. This large gap in the cost of repatriation indicates a great heterogeneity in repatriation cases. This variance was driven by large variations in the average direct costs. These differences were mainly due to large differences in; *I) the costs of evacuation (getting the seafarer from the vessel to shore) and II) differences in treatment costs.* The variations in indirect costs between the cases were mainly due to differences in the replacement costs. The average indirect costs varied between 14,000 euro in the malaria case and almost 22,000 euro in the malignant hypertension case vignette. The variation indicates that average indirect cost proportions ranges between 22 and 60% in the case vignettes. Indirect costs remain a substantial cost driver and outline the importance of estimating indirect costs, when estimating the total costs of repatriation.

In the AMI case vignette the data revealed, that the employer had costs which were not reimbursed by DMA and the estimate of 98,823 euro is, therefore, likely to be an underestimate of the financial burden held by the employer in this repatriation case. This illustrates that using the data from DMA as a proxy for the direct costs held by the employer is not entirely accurate.

Previous data suggests that the annual number of repatriations amounts to nearly 10,000 with an annual cost of 760 million euro [2]. With the costs per repatriation case ranging between 24,000 and 99,000 euro the annual cost of 10,000 repatriations cases is somewhere between 240 million and 1 billion euros.

Results from similar studies using the case vignette approach to estimate the costs of AMI treatment in nine selected countries under DRG tariffs showed big differences among countries too. In France, The Netherlands and Italy, PCI was the standard of care intervention to treat AMI, with costs ranging between 3720 and 9374 euros [28]. In case vignette number one, the corresponding treatment for an AMI was PCI with a cost of nearly 43,000 euro, which makes the treatment of this case vignette much higher than the estimates provided by the EU HealthBASKET project [28]. Similar studies found AMI treatment costs to range between 5434 and 7770 euros [29, 30].

Similar results were found with regards to the costs of treatment for appendicitis [18]. The mean total costs per case vignette across the selected countries were 1601 euro. Spain, was included in the selected countries of the EU HealthBASKET project, had a very low cost of treating appendicitis at a mean of 594 euro in DGR tariffs [31]. However, the findings of this survey showed the treatment costs for the case vignette number three were 10,076 euro, for the same country. Only in the United States, the most expensive health care system in the world treatment of appendectomy [32], was found to be similar to those found in this study [33].

This implies that in the case of seafarers – international employees not covered by any health system – market prices are used for the health services provided to them, contributing to a higher treatment cost.

The cost of helicopter evacuation at sea was estimated to be 25,000 euros per intervention [2]. Helicopter evacuation at sea has some similarities with remote area helicopter emergency services, which have average costs per mission varying between 6600 and 13,500 euros [34, 35].

A considerable amount of indirect cost represented the ship deviations depending on the distance from the nearest shore.

Any model is always a simplification of the reality [36], and since the available data did not cover all aspects of the model, several assumptions had to be made and assumptions come with uncertainty. In order to produce a robust cost analysis, special attention should be made in regards to properly estimate the major cost drivers [19]. The three major cost drivers for repatriation were transport from vessel to shore either by helicopter or ship deviation, treatment, and sickness benefits. Sickness benefits and treatment costs were reimbursed and are in the DMA records, which is why these estimates are fairly valid. The salary of the seafarers and transportation from sea to shore and insurance premium increase are all based on published literature and assumptions since no local level data were available and therefore they are surrounded by some degree of uncertainty. The companies should seek to routinely collect and make available these data.

This study did not estimate any costs in terms of charterer loss penalties, which could imply that estimates of repatriation in the current study are conservative estimates of the true total costs.

By applying the case vignette approach, it was assumed that cases in each case vignette represents the typical repatriation case of this disease, however a more representative sample could provide more accurate cost data.

## Conclusion

This study was a first attempt to map the relevant financial burden to the employer due to employee sickness on board merchant vessels. The objective was to estimate the costs of repatriation of ill seafarers based on four case vignettes; *I) acute myocardial infarction, II) malignant hypertension, III) acute appendicitis and IV) malaria.* The findings are a framework for investigating the average total costs of repatriation by applying a local level micro-costing approach. The cost formula included direct and indirect costs and the local approach proved to be a feasible approach to estimate the total costs of repatriation with an employer perspective.

Every case of repatriation poses a large financial burden on employers, and the results indicated a large variation in the average total costs of repatriation for the four case vignettes. These variations in the proportions of direct cost were mainly due to hospitalization and deviation expenses. It is worth noticing that fee-for-service contributed to higher prices for treating seafarers around the globe. This clearly shows how prices for treating the same diagnosis differentiate in different countries and give insight for introducing possible collective bargaining agreements.

With regards to the indirect costs the recruitment of a new employee was the main cost driver among the case vignettes. In our analysis, it was established that indirect costs are an important estimate from the employer’s point of view, and a major cost driver for the total costs of repatriation. These indirect costs are not reimbursed, and they all fall directly upon the employer. In order to estimate the total costs of repatriation, it would be beneficial if these cost data were collected regularly at the company level.

From the employers’ point of view, it would be interesting to know these costs of repatriation, especially regarding helicopter evacuation, deviation and charterer loss. This could help the employers’ insight and motivation for disease preventive and health promotion interventions on board.

Employers have a financial interest in promoting the health of seafarers by introducing or strengthening cost-effective prevention and health promotion programs, and hereby reducing the number of repatriations, as each repatriation of an ill seafarer is a heavy financial burden to the employer.

## Abbreviations

AMI: Acute myocardial infarction
DKK: Danish Kroner
DMA: Danish Maritime Authority
EU: European Union
EUR: Euro
ITF: International Transport Workers Federation
MLC: Maritime Labour Convention
PCI: Percutaneous coronary intervention

## Footnotes

1 Evacuation is a process of transporting the seafarer from the vessel to the nearest suitable port for medical treatment with repatriation be further transportation on to the home country, where additional treatment may be needed [9].
